# A Real-World, Population-Based Retrospective Analysis of Therapeutic Survival for Recurrent Localized Renal Cell Carcinoma After Nephrectomy

**DOI:** 10.3389/fonc.2021.693831

**Published:** 2021-09-08

**Authors:** Sung Han Kim, Min Gee Choi, Ji Hye Shin, Young-Ae Kim, Jinsoo Chung

**Affiliations:** ^1^Department of Urology, Center for Urologic Cancer, Research Institute and Hospital of National Cancer Center, Goyang, South Korea; ^2^National Cancer Control Institute, National Cancer Center, Goyang, South Korea

**Keywords:** renal cell carcinoma, nephrectomy, treatment, recurrence, prognosis

## Abstract

We retrospectively analyzed therapeutic strategies and risk factors for overall survival (OS) in disease recurrence following curative nephrectomy for localized renal cell carcinoma (loRCC) using the Korean National Cancer Registry Database. We selected 1295 recurrent loRCC patients who underwent either partial or radical nephrectomy from 2007–2013. Patients were excluded for age <19 years, secondary RCC, multiple primary tumors, other SEER stages except for a localized or regional stage, postoperative recurrence within 3-month, and non-nephrectomized cases. Four therapeutic groups were statistically analyzed for OS and risk factors: surgery (OP, 12.0%), other systemic therapy (OST, 59.5%), radiotherapy (RT, 2.8%), and targeted therapy (TT, 25.8%). The overall mortality rate for recurrent loRCC was 32.5%, including 82.4% for RCC-related deaths. The baseline comparison among groups showed statistical differences for the diagnostic age of cancer and the SEER stage (p<0.05). Multivariate analysis of OS showed significance for the TT (hazard ratio [HR]: 6.27), OST (HR: 7.05), and RT (HR: 7.47) groups compared with the OP group, along with significance for the sex, SEER stage, and the time from nephrectomy to treatment for disease recurrence (p<0.05). The median OS curve showed a significantly better OS in the OP group (54.9 months) compared with the TT, OST, and RT groups (41.7, 42.9, and 38.0 months, respectively; p<0.001). In conclusion, the surgery-treated group had the best OS among the different therapeutic strategies for recurrent loRCC after nephrectomy, and the importance of the time from nephrectomy to secondary treatment was a significant prognostic factor.

## Introduction

Renal cell carcinoma (RCC) is a radio- and chemo-resistant tumor for which surgical removal of primary kidney tumor *via* radical or partial nephrectomy in localized RCC (loRCC) is the standard curative strategy (1, 2). Once the RCC becomes either advanced or metastatic, the 5-year overall survival (OS) rates significantly decrease from 85–95% to <30% for loRCC (3). Among the surgically treated loRCC cases, about 33–50% of patients experience disease recurrence within two years resulting in a survival rate of <30% (1, 4–7), with rarely later recurrence after postoperative 5-year (6, 8–10). Therefore, it is important to identify any predisposing characteristics of disease recurrence after nephrectomy and detect them as early as possible to initiate an earlier therapeutic strategy with adequate periodic imaging methods to improve the survival prognosis.

The recurrent disease includes distant metastasis and local disease recurrence. Several therapeutic modalities are available for its treatment, dependent on tumor-related risk factors, the intrinsic tumor pathology, and the extent and location of the recurrence; this results in diverse and unpredictable therapeutic outcomes (5, 6). In addition to there being multiple therapeutic priorities, deciding the most appropriate and best therapeutic options for disease recurrence is also complicated by its rarity, unpredictability, tumor heterogeneity, and the lack of large-scale, randomized studies that support any management type (1, 6–8). Since the disease recurrence can be a local recurrence at the operative area, distant metastasis, or synchronous local recurrence and distant metastasis, various and complex therapeutic strategies exist in combination with local and systemic therapies.

Due to the differences, heterotrophic heterogenicity, and complex appearances of local recurrence from distant metastasis, it is difficult to determine the most appropriate treatment and to generalize recommendations from randomized trials for improving survival prognoses and treatment outcomes (4–6, 8). This creates uncertainty in real-world clinics for choosing the best treatment for recurrent loRCC following a nephrectomy based on information from the national insurance coverage system, clinicians’ experiences, and a few multicentric, retrospective, and prospective studies (1, 4–8).

This study retrospectively evaluated, for the first time in Korea, the therapy methods and patterns of recurrent loRCC after either radical or partial nephrectomy using a population-based National Cancer database and the National Insurance Claim databases in Korea to analyze overall survival (OS) and related risk factors.

## Materials and Methods

### Ethics Statement

This study was approved by the Institutional Review Board (IRB) of the National Cancer Center and Cancer Research Institute in Korea (IRB no. NCC2015-0217). The analytical methodology for using the Korean National Policy System database has been previously described (10). A detailed cohort was selected for cancer incidence and mortality data from 2006–2013 from the Korean National Health Insurance System of Statistics and the Korea National Cancer Incidence Database of Korean Central Cancer Registry.

### Patients’ Criteria

**Figure 1:**
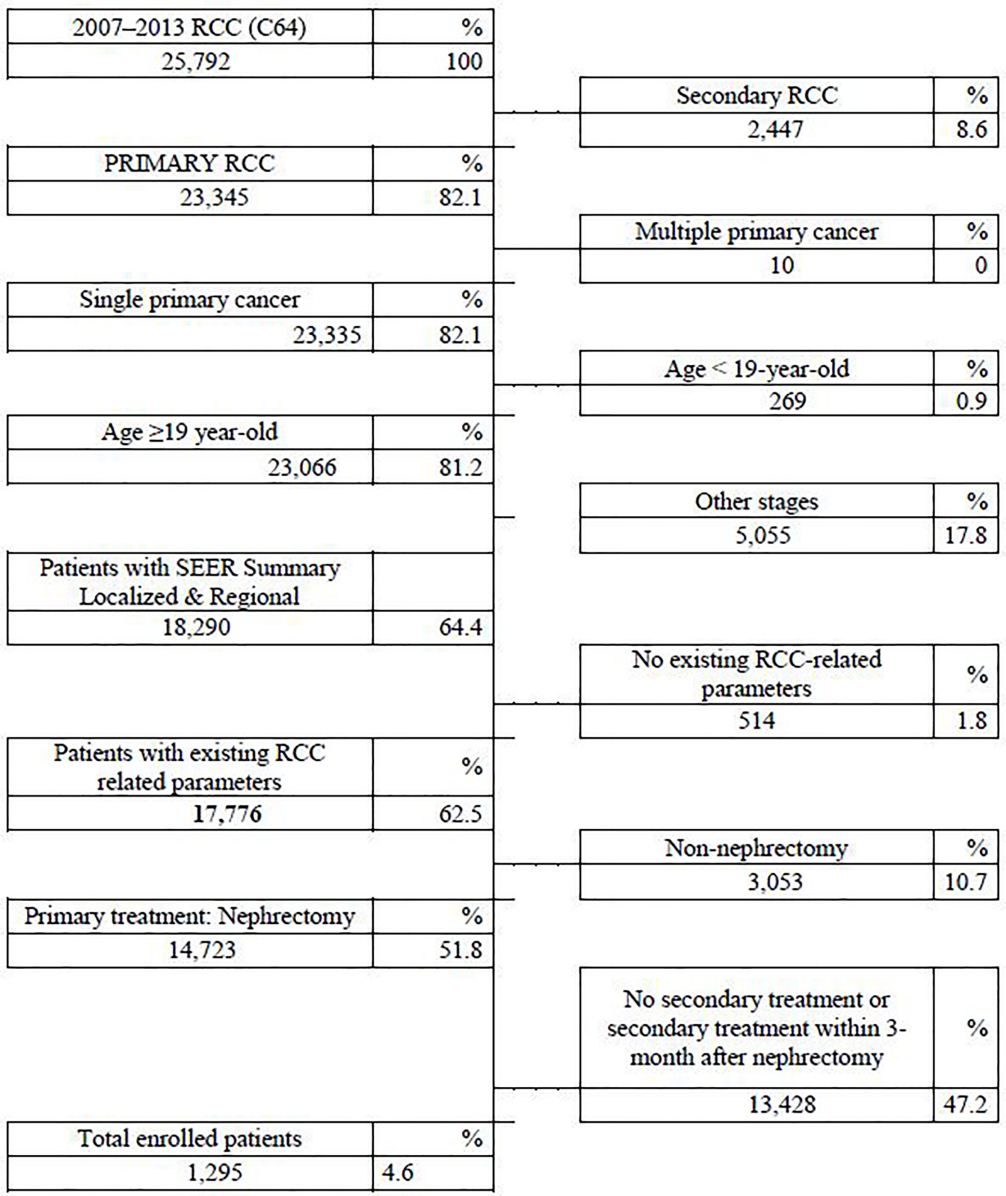
Flowchart of patient selection according to inclusion and exclusion criteria in the National Insurance Policy Database.

An ICD-10 code of C64 for RCC from the Korean National Insurance Policy Database was used to extract data for patients with localized RCC following either partial or radical nephrectomy who had postoperative secondary treatment from 2007–2013. LoRCC was defined as a localized or regional stage according to the SEER staging system. Patients were excluded for age <19 years, secondary RCC, multiple primary tumors, other SEER stages except for localized and regional, postoperative therapies within 3 months after nephrectomy, and non-nephrectomized cases. There were 1295 (4.6%) nephrectomy cases selected out of 25,792 cases **Figure 1**.

#### Group Definitions According to the Secondary Therapies

The therapeutic treatments 3-months after nephrectomy for the 1295 patients were grouped as OP for surgery (n=155, 12.0%), OST for other systemic therapy (n=334, 25.8%), RT for radiation therapy (n=36, 2.8%), and TT for targeted therapy (n=770, 59.5%) according to the first method of secondary treatment after nephrectomy. For example, the patient was enrolled into the OP group if the recurrent loRCC received surgery and adjuvant or neoadjuvant chemotherapy, radiation therapy, or targeted therapy. However, when the patient received only TT as the first therapy, the patient was enrolled into the TT group. When the patient received OST first and then either TT or RT, the patient was enrolled in the OST group. This means the RT group was composed of patients that received RT only. We determined the postoperative 3-month time index to exclude the obscured synchronous metastatic RCC at nephrectomy and include the post-nephrectomized recurrent LoRCC.

### Statistical Analysis

Student’s t-test, the chi-square test, and Fisher’s exact test were used to compare the baseline characteristics between groups, including age at RCC diagnosis, sex, residential location, insurance type, SEER stage, year of RCC diagnosis, time from cancer diagnosis to the first nephrectomy, time from nephrectomy to the secondary treatment for disease recurrence, secondary treatment type, and mortality/survival (**Table 1**). The OS of the four therapeutic groups were compared. Multivariate analysis was performed for the risk factors of OS, and Kaplan-Meier analysis with the log-rank test were performed; a p-value <0.05 was considered significant. From the starting date of nephrectomy, hazard ratios (HR) with 95% confidence interval (CI) values from Cox proportional hazard models for mortality were analyzed to investigate the effects of secondary treatments. Age at RCC diagnosis, sex, residential location, insurance type, SEER stage, year of RCC diagnosis, time from cancer diagnosis to the first nephrectomy, and time from nephrectomy to the secondary treatment for disease recurrence were used to adjust the Cox proportional model. Two-sided p-values <0.05 were considered statistically significant. All statistical analyses were performed using SAS (release 9.4, SAS Institute Inc., North Carolina, USA).

**Table 1:** Overall patient’s baseline demographics according to the therapeutic types.

	Secondary treatment for fist OP										p
	Total (n = 1,295)		OP (n = 155)		TT (n = 774)		OST (n = 330)		RT (n = 36)		
	n	%	n	%	n	%	n	%	n	%	
**Sex**											
** Male**	995	76.8	121	78.1	602	77.8	244	74.9	28	77.8	0.554
** Female**	300	23.2	34	21.9	172	22.2	86	26.1	8	22.2	
**Age at cancer diagnosis, y**											
** 19–39**	73	5.6	27	17.4	38	4.9	8	2.4	0	0.0	<0.0001
** 40–49**	180	13.9	36	23.2	111	14.3	27	8.2	6	16.7	
** 50–59**	352	27.2	52	33.6	220	28.4	68	20.6	12	33.3	
** 60–69**	391	30.2	33	21.3	243	31.4	106	32.1	9	25.0	
** 70+**	299	23.1	7	4.5	162	20.9	121	36.7	9	25.0	
**Residence**											
** Rural**	670	51.7	89	57.4	390	50.4	178	53.9	13	36.1	0.081
** Urban**	625	48.3	66	42.6	384	49.6	152	46.1	23	63.9	
**BMI**											
** Underweight (<18.6)**	26	2.0	3	3.3	14	3.3	9	4.8	0	0.0	0.874
** Normal(18.6=<BMI<25.0)**	391	30.2	47	52.2	235	55.7	100	53.5	9	50.0	
** Obese(25.0=<BMI<30.0)**	271	20.9	35	38.9	160	37.9	68	36.4	8	44.4	
** Very obese(30.0=<BMI)**	29	2.2	5	5.6	13	3.1	10	5.4	1	5.6	
** Missing**	578	44.6									
**Comorbidities**											
**HTN**	1009	77.9	116	74.8	605	78.2	260	78.8	28	77.8	0.795
**DM**	771	59.5	92	59.3	453	58.5	203	61.5	23	63.9	0.765
**Cardiovascular disease**	457	35.3	59	38.1	258	33.3	121	36.7	19	52.8	0.075
**Cerebrovascular disease**	304	23.5	25	16.1	177	22.9	95	28.8	7	19.4	0.016
**CCI**											
**0**	433	33.4	49	31.6	287	37.1	85	25.8	12	33.3	0.012
**1**	263	20.3	35	22.6	153	19.8	64	19.4	11	30.6	
**2**	264	20.4	35	22.6	150	19.4	74	22.4	5	13.9	
**3=<**	335	25.9	36	23.2	184	23.8	107	32.4	8	22.2	
**Histology**											
**Non-clear cell**	29	2.2	2	1.3	8	1.0	17	5.2	2	5.6	0.0001
**Clear cell**	1266	97.8	153	98.7	766	99.0	313	94.9	34	94.4	
**Insurance**											
**Occupational**	796	61.5	100	64.5	467	60.3	206	62.4	23	63.9	0.461
**Regional**	427	33.0	42	27.1	267	34.5	106	32.1	12	33.3	
**Medicare**	72	5.6	13	8.4	40	5.2	18	5.5	1	2.8	
**Seer Summary stage**											
**Localized**	859	66.3	142	91.6	453	58.5	243	73.6	21	58.3	<0.0001
**Regional**	436	33.7	13	8.4	321	41.5	87	26.4	15	41.7	
**Year of cancer diagnosis**											
**2007**	195	15.1	23	14.8	110	14.2	57	17.3	5	13.9	0.590
**2008**	196	15.1	22	14.2	114	14.7	55	16.7	5	13.9	
**2009**	186	14.4	26	16.8	99	12.8	56	17.0	5	13.9	
**2010**	220	17.0	23	14.8	148	19.1	43	13.0	6	16.7	
**2011**	183	14.1	24	15.5	104	13.4	50	15.2	5	13.9	
**2012**	174	13.4	20	12.9	114	14.7	36	10.9	4	11.1	
**2013**	141	10.9	17	11	85	11.0	33	10.0	6	16.7	
**Time from cancer diagnosis to first op**											
**0 days**	433	33.4	59	38.1	250	32.3	114	34.6	10	27.8	0.378
**1–7 days**	161	12.4	19	12.3	102	13.2	38	11.5	2	5.6	
**8–14 days**	181	14.0	23	14.8	114	14.7	36	10.9	8	22.2	
**15–21 days**	266	20.5	25	16.1	164	21.2	67	20.3	10	27.8	
**30+ days**	254	19.6	29	18.7	144	18.6	75	22.7	6	16.7	
**Time from nephrectomy to the secondary treatment for disease recurrence**											
**~9 months**	347	26.8	38	24.5	207	26.7	92	27.9	10	27.8	0.466
**10–11 months**	64	4.9	3	1.9	42	5.4	15	4.6	4	11.1	
**12–23 months**	314	24.2	34	21.9	185	23.9	86	26.1	9	25	
**24–35 months**	209	16.1	25	16.1	128	16.5	51	15.5	5	13.9	
**36+ months**	361	27.9	55	35.5	212	27.4	86	26.1	8	22.2	
**Mortality**											
**Alive**	874	67.5	147	94.8	508	65.6	196	59.4	23	63.9	<0.0001
**Death**	421	32.5	8	5.2	266	34.4	134	40.6	13	36.1	
**Cause of death**											
**Cancer cause death**	347	82.4	6	75	245	92.1	83	61.9	13	100	<0.0001
**Other cancer cause death**	47	11.2	0	0	12	4.5	35	26.1	0		
**Other cause death**	27	6.4	2	25	9	3.4	16	11.9	0		

OP, surgery; TT, targeted therapy; OS, other systemic agent therapy besides the targeted therapy; RT, radiotherapy.

## Results

### Baseline Characteristics

**Figure 2:**
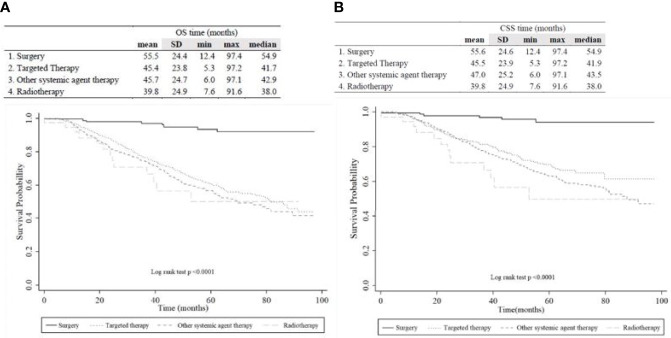
Kaplan-Meier plot of **(A)** overall survival and **(B)** cancer-specific survival among overall patients according to the therapeutic group.

Among 1124 radically nephrectomized patients and 171 partially nephrectomized patients, 421 (32.5%) deaths were observed during the study period, including 17.6% of non-RCC-specific deaths (**Table 1**). The median OS of each group was 54.9, 41.7, 42.9, and 38.0 months for the OP, TT, OST, and RT groups, respectively (**Figure 2**). The median periods for each treatment group from nephrectomy to the secondary treatment after postoperative disease progression were 32.7, 26.0, 25.2, and 24.1 months for the OP, TT, OST, and RT groups, respectively.

The comparison of baseline characteristics between the four treatment groups showed that the cancer-diagnostic age, the presence of cerebrovascular disease, the CCI, the histology, the SEER stage, and the mortality and survival rates were significantly different (p<0.05, **Table 1**). The comparison of baseline characteristics between the radical and partially nephrectomized groups showed that only the SEER stage and mortality/survival rates were significantly different (p<0.001, **Supplementary Table 1**). Among the radically nephrectomized group (n=1124, **Table 2**), the comparison of baseline characteristics among the four different therapeutic groups showed results similar to those in **Table 1**: significant differences were observed in the cancer-diagnostic age, the presence of cardiovascular disease, the histology, the SEER stage, and the mortality and survival rates (p<0.05).

**Table 2:** Baseline characteristics among 1124 radically nephrectomized patients.

	Secondary treatment for disease recurrence after radical nephrectomy								p
	OP (n = 97)		TT (n = 733)		OST (n = 265)		RT (n = 29)		
	n	%	n	%	n	%	n	%	
Sex									
Male	72	74.2	570	77.8	191	72.1	23	79.3	0.2805
Female	25	25.8	163	22.2	74	27.9	6	20.7	
Age at cancer diagnosis, y									
19–39	18	18.6	35	4.8	8	3.0	0	0.0	<0.0001
40–49	28	28.9	109	14.9	22	8.3	4	13.8	
50–59	31	32.0	208	28.4	56	21.1	10	34.5	
60–69	17	17.5	226	30.8	83	31.3	8	27.6	
70+	3	3.1	155	21.2	96	36.2	7	24.1	
Residence									
Rural	59	60.8	371	50.6	137	51.7	10	34.5	0.0732
Urban	38	39.2	362	49.4	128	48.3	19	65.5	
BMI *Missing (n=522)									
Underweight	3	5.9	14	3.5	8	5.7	0	0.0	0.7035
Normal	25	49.0	221	55.8	73	51.8	6	42.9	
Obese	20	39.2	149	37.6	52	36.9	7	50.0	
Very obese	3	5.9	12	3.0	8	5.7	1	7.1	
Comorbidities									
HTN	80	82.5	570	77.8	211	79.6	23	79.3	0.7184
DM	69	71.1	428	58.4	162	61.1	20	69.0	0.0765
Cardiovascular disease	43	44.3	241	32.9	94	35.5	17	58.6	0.0065
Cerebrovascular disease	13	13.4	166	22.7	71	26.8	6	20.7	0.0614
CCI									
0	25	25.8	275	37.5	76	28.7	11	37.9	0.0749
1	26	26.8	147	20.1	52	19.6	7	24.1	
2	21	21.7	141	19.2	52	19.6	5	17.2	
3	25	25.8	170	23.2	85	32.1	6	20.7	
Histology									
Non-clear cell	2	2.1	7	1.0	16	6.0	2	6.9	<0.0001
Clear cell	95	97.9	726	99.1	249	94.0	27	93.1	
Insurance									
Occupational	61	62.9	441	60.2	163	61.5	19	65.5	0.4684
Regional	26	26.8	252	34.4	85	32.1	9	31	
Medicare	10	10.3	40	5.5	17	6.4	1	3.5	
Seer Summary stage									
Localized	88	90.7	414	56.5	181	68.3	15	51.7	<0.0001
Regional	9	9.3	319	43.5	84	31.7	14	48.3	
Year of cancer diagnosis									
2007	16	16.5	106	14.5	48	18.1	4	13.8	0.3694
2008	12	12.4	112	15.3	46	17.4	4	13.8	
2009	19	19.6	94	12.8	45	17.0	4	13.8	
2010	16	16.5	137	18.7	31	11.7	5	17.2	
2011	15	15.5	98	13.4	42	15.9	4	13.8	
2012	9	9.3	107	14.6	31	11.7	3	10.3	
2013	10	10.3	79	10.8	22	8.3	5	17.2	
Time from cancer diagnosis to first op									
0 days	38	39.2	241	32.9	93	35.1	7	24.1	0.2221
1-7 days	12	12.4	99	13.5	30	11.3	0	0.0	
8-14 days	14	14.4	107	14.6	29	10.9	6	20.7	
15-21 days	17	17.5	152	20.7	52	19.6	10	34.5	
30+ days	16	16.5	134	18.3	61	23.0	6	20.7	
Time from nephrectomy to the secondary treatment for disease recurrence									
~ 9 months	19	19.6	201	27.4	73	27.6	9	31.0	0.3648
10–11 months	2	2.1	41	5.6	14	5.3	3	10.3	
12–23 months	23	23.7	175	23.9	71	26.8	8	27.6	
24–35 months	15	15.5	119	16.2	40	15.1	3	10.3	
36+ months	38	39.2	197	26.9	67	25.3	6	20.7	
Mortality									
Alive	91	93.8	474	64.7	151	57.0	18	62.1	<0.0001
Death	6	6.2	259	35.3	114	43.0	11	37.9	
Cause of death *Missing (n=734)									
Cancer cause death	4	66.7	239	92.3	75	65.8	11	100	<0.0001
Other cancer cause death	0	0.0	11	4.3	25	21.9	0	0.0	
Other cause death	2	33.3	9	3.5	14	12.3	0	0.0	

OP, surgery; TT, targeted therapy; OST, other systemic agent therapy besides the targeted therapy; RT, radiotherapy.

### Multivariate Results for Survival Outcomes

In the multivariate analysis of OS, the obese BMI (HR: 0.47, 95%CI: 0.23–0.96), regional SEER stage (HR: 1.78, 95%CI: 1.29–2.47), a postoperative after-two-year from nephrectomy to secondary treatment for disease recurrence (HR: <1.0, years post-nephrectomy), and the secondary treatment types (HR: 7.62 for TT and HR 7.68 for OST) were significant risk factors (p<0.05, **Table 3**). In adjusted multivariate analysis, only 1124 radically nephrectomized RCC patients had predictive OS risk factors similar to 1295 overall patients (p<0.05, **Supplementary Table 2**).

**Table 3:** Multivariate analysis of overall survival in RCC patients with postoperative disease progression after nephrectomy.

	Univariate				Multivariate			
	HR	95% CI		p	HR	95% CI		p
Sex								
Male	1							
Female	0.92	0.73	1.16	0.464				
Age at cancer diagnosis, y								
19–39	1				1			
40–49	1.08	0.65	1.78	0.7806	1.33	0.43	4.14	0.623
50–59	0.86	0.54	1.39	0.548	0.96	0.32	2.86	0.939
60–69	1.39	0.88	2.19	0.1626	1.60	0.55	4.67	0.390
70+	1.81	1.14	2.88	0.0125	1.92	0.64	5.78	0.245
Residence								
Rural	1							
Urban	1.05	0.87	1.27	0.613				
BMI								
Underweight	1				1			
Normal	0.63	0.33	1.21	0.163	0.61	0.30	1.21	0.156
Obese	0.48	0.25	0.93	0.031	0.47	0.23	0.96	0.038
Very obese	0.25	0.09	0.88	0.029	0.37	0.12	1.21	0.101
Comorbidities								
HTN	0.79	0.77	1.23	0.8285				
DM	1.17	0.96	1.43	0.1311				
Cardiovascular disease	0.82	0.67	1.01	0.0562				
Cerebrovascular disease	1.05	0.85	1.31	0.6336				
CCI								
0	1				1			
1	1.23	0.94	1.61	0.126	1.34	0.87	2.05	0.181
2	1.11	0.84	1.48	0.465	1.06	0.67	1.68	0.811
3	1.39	1.09	1.79	0.009	1.36	0.91	2.04	0.139
Histology								
Non-clear cell	1				1			
Clear cell	0.49	0.30	0.82	0.006	0.80	0.28	2.26	0.676
Seer Summary stage								
Localized	1				1			
Regional	2.17	1.79	2.64	<0.0001	1.78	1.29	2.47	0.001
Year of cancer diagnosis								
2007	1							
2008	1.08	0.80	1.47	0.614				
2009	1.14	0.83	1.57	0.410				
2010	1.15	0.83	1.60	0.388				
2011	1.22	0.84	1.77	0.288				
2012	1.40	0.92	2.13	0.116				
2013	1.40	0.77	2.55	0.264				
Time from cancer diagnosis to nephrectomy								
0 days	1							
1–7 days	0.92	0.67	1.27	0.616				
8–14 days	0.85	0.61	1.17	0.319				
15–21 days	0.93	0.71	1.22	0.602				
30+ days	1.01	0.78	1.32	0.928				
Time from nephrectomy to the secondary treatment for disease recurrence								
~9 months	1				1			
10–11 months	1.11	0.77	1.61	0.583	0.97	0.49	1.92	0.921
12–23 months	0.68	0.54	0.87	0.002	0.81	0.54	1.21	0.302
24–35 months	0.38	0.28	0.51	<0.0001	0.44	0.27	0.71	0.001
36+ months	0.13	0.10	0.179	<0.0001	0.18	0.11	0.28	<0.0001
Nephrectomy type								
Partial nephrectomy	1				1			
Radical nephrectomy	2.09	1.45	3.01	<0.0001	1.16	0.67	2.01	0.587
Second treatment type								
Surgery	1				1			
Targeted therapy	8.44	4.17	17.05	<0.0001	7.62	2.36	24.59	0.001
Other systemic agent therapy	9.88	4.84	20.18	<0.0001	7.68	2.37	24.94	0.001
Radiotherapy	10.43	4.32	25.16	<0.0001	4.68	0.93	23.64	0.062

In the multivariate analysis of CSS, the regional SEER stage (HR: 1.83, 95%CI: 1.28–2.62), a postoperative after-two-year from nephrectomy to secondary treatment for disease recurrence (HR: <1.0, years post-nephrectomy), and the secondary treatment types (HR: 9.86 for TT, HR 7.77 for OST, and HR 8.01 for RT) were significant risk factors (p<0.05, **Table 4**).

**Table 4:** Multivariate analysis of RCC specific survival in RCC patients with postoperative disease progression after nephrectomy.

	Univariate				Multivariate			
	HR	95% CI		p	HR	95% CI		p
Sex								
Male	1							
Female	0.91	0.71	1.18	0.486				
Age at cancer diagnosis, y								
19–39	1							
40–49	1.03	0.62	1.74	0.899				
50–59	0.76	0.46	1.25	0.279				
60–69	1.20	0.74	1.92	0.460				
70+	1.37	0.84	2.23	0.203				
Residence								
Rural	1							
Urban	1.00	0.81	1.23	0.962				
BMI								
Underweight	1							
Normal	0.63	0.31	1.31	0.219				
Obese	0.48	0.23	1.02	0.055				
Very obese	0.35	0.10	1.15	0.083				
Comorbidities								
HTN	0.91	0.71	1.18	0.475				
DM	1.06	0.85	1.32	0.621				
Cardiovascular disease	0.79	0.64	0.99	0.041	0.87	0.61	1.23	0.431
Cerebrovascular disease	0.93	0.72	1.19	0.544				
CCI								
0	1							
1	1.21	0.90	1.62	0.200				
2	1.08	0.79	1.47	0.642				
3	1.26	0.96	1.66	0.094				
Histology								
Non-clear cell	1							
Clear cell	0.74	0.38	1.43	0.363				
Seer Summary stage								
Localized	1				1			
Regional	2.38	1.93	2.95	<0.0001	1.83	1.28	2.62	0.001
Year of cancer diagnosis								
2007	1							
2008	1.06	0.75	1.50	0.728				
2009	1.16	0.82	1.65	0.409				
2010	1.21	0.85	1.73	0.298				
2011	1.26	0.84	1.89	0.263				
2012	1.50	0.96	2.35	0.073				
2013	1.42	0.74	2.70	0.291				
Time from cancer diagnosis to first op								
0 days	1							
1–7 days	0.90	0.63	1.29	0.578				
8–14 days	0.92	0.65	1.31	0.657				
15–21 days	0.94	0.70	1.27	0.702				
30+ days	1.01	0.76	1.35	0.939				
Time from nephrectomy to the secondary treatment for disease recurrence								
~9 months	1				1			
10–11 months	1.09	0.73	1.63	0.671	0.95	0.46	1.95	0.884
12–23 months	0.65	0.50	0.84	0.001	0.69	0.45	1.06	0.092
24–35 months	0.32	0.22	0.45	<0.0001	0.42	0.25	0.73	0.002
36+ months	0.13	0.09	0.18	<0.0001	0.19	0.12	0.32	<0.0001
Nephrectomy type								
Partial nephrectomy	1				1			
Radical nephrectomy	3.02	1.88	4.86	<0.0001	1.48	0.75	2.96	0.262
Second treatment type								
Surgery	1				1			
Targeted therapy	10.25	4.56	23.04	<0.0001	9.86	2.38	40.82	0.002
Other systemic agent therapy	8.09	3.53	18.54	<0.0001	7.77	1.86	32.56	0.005
Radiotherapy	13.70	5.21	36.07	<0.0001	8.01	1.32	48.60	0.024

### Propensity Score Matching Results

The three different treatment groups were compared after 1:1 Propensity-score matching by adjusting the gender, age, CCI, and SEER staging. In the comparison between the OP and TT groups, the presence of comorbid diseases such as hypertension and diabetes, mortality and cause of death were significantly different (p<0.05, **Table 5**). In comparing TT and OST groups, histology, year of cancer diagnosis, and cause of death were significantly different (p<0.05).

**Table 5:** PS matching 1:1 adjusted by matching variable: gender, SEER staging, CCI, and age.

	Secondary treatment for disease recurrence after radical nephrectomy									p
	OP		TT		p	TT		OST		
	(n = 97)		(n = 97)			(n = 265)		(n = 265)		
	n		n			n		n	%	
Sex										
Male	72	74.23	72	74.2	1	187	70.6	191	72.1	0.7008
Female	25	25.77	25	25.8		78	29.4	74	27.9	
Age at cancer diagnosis, yea										
19–39	18	18.56	16	16.5	0.9959	8	3.0	8	3.0	0.9999
40–49	28	28.87	30	30.9		21	7.9	22	8.3	
50–59	31	31.96	31	32.0		56	21.1	56	21.1	
60–69	17	17.53	17	17.5		84	31.7	83	31.3	
70+	3	3.09	3	3.1		96	36.2	96	36.2	
Residence										
Rural	59	60.8	49	50.5	0.1484	133	50.2	137	51.7	0.7282
Urban	38	39.2	48	49.5		132	49.8	128	48.3	
BMI										
Underweight	3	5.9	4	6.9	0.9818	7	4.9	8	5.7	0.2572
Normal	25	49	30	51.7		80	55.6	73	51.8	
Obese	20	39.2	21	36.2		55	38.2	52	36.9	
Very obese	3	5.9	3	5.2		2	1.4	8	5.7	
Comorbidities										
HTN	80	82.5	66	68.0	0.0198	217	81.9	211	79.6	0.5085
DM	69	71.1	55	56.7	0.0363	151	57.0	162	61.1	0.3312
Cardiovascular disease	43	44.3	31	32.0	0.0761	80	30.2	94	35.5	0.1953
Cerebrovascular disease	13	13.4	11	11.3	0.6628	66	24.9	71	26.8	0.6198
CCI										
0	25	25.8	27	27.8	0.6804	76	28.7	76	28.7	0.9279
1	26	26.8	22	22.7		53	20.0	52	19.6	
2	21	21.7	27	27.8		57	21.5	52	19.6	
3	25	25.8	21	21.7		79	29.8	85	32.1	
Histology										
Non-clear cell	2	2.1	2	2.1	1	3	1.1	16	6.0	0.0024
Clear cell	95	97.9	95	97.9		262	98.9	249	94.0	
Insurance										
Occupational	61	62.9	50	51.6	0.0384	154	58.1	163	61.5	0.4096
Regional	26	26.8	42	43.3		86	32.5	85	32.1	
Medicare	10	10.3	5	5.2		25	9.4	17	6.4	
Seer Summary stage										
Localized	88	90.7	88	90.7	1	178	67.2	181	68.3	0.7804
Regional	9	9.3	9	9.3		87	32.8	84	31.7	
Year of cancer diagnosis										
2007	16	16.5	14	14.4	0.1188	24	9.1	48	18.1	0.0055
2008	12	12.4	28	28.9		53	20.0	46	17.4	
2009	19	19.6	12	12.4		31	11.7	45	17.0	
2010	16	16.5	11	11.3		47	17.7	31	11.7	
2011	15	15.5	13	13.4		36	13.6	42	15.9	
2012	9	9.3	12	12.4		44	16.6	31	11.7	
2013	10	10.3	7	7.2		30	11.3	22	8.3	
Time from cancer diagnosis to first op										
0 days	38	39.2	35	36.1	0.9486	95	35.9	93	35.1	0.601
1-7 days	12	12.4	15	15.5		38	14.3	30	11.3	
8-14 days	14	14.4	12	12.4		35	13.2	29	10.9	
15-21 days	17	17.5	19	19.6		45	17.0	52	19.6	
30+ days	16	16.5	16	16.5		52	19.6	61	23.0	
Time from nephrectomy to the secondary treatment for disease recurrence										
~9 months	19	19.6	18	18.6	0.9787	78	29.4	73	27.6	0.9892
10–11 months	2	2.1	2	2.1		13	4.9	14	5.3	
12–23 months	23	23.7	24	24.7		68	25.7	71	26.8	
24–35 months	15	15.5	18	18.6		41	15.5	40	15.1	
36+ months	38	39.2	35	36.1		65	24.5	67	25.3	
Mortality										
Alive	91	93.8	67	69.1	<0.001	169	63.8	151	57.0	0.1099
Death	6	6.2	30	30.9		96	36.2	114	43.0	
Cause of death Missing										
Cancer cause death	4	66.7	29	96.7	0.0152	88	91.7	75	65.8	<0.001
Other cancer cause death	0	0	0	0.0		4	4.2	25	21.9	
Other cause death	2	33.3	1	3.3		4	4.2	14	12.3	

OP, surgery; TT, targeted therapy; OST, other systemic agent therapy besides the targeted therapy; RT, radiotherapy.

In the multivariate analysis of CSS with 1:1 propensity score matching cohort between OP and TT groups (**Table 6A**), the time from nephrectomy to the secondary treatment since 2 years (24-35 months, HR 0.24 CI 0.073-0.784; 36-month< HR 0.124, CI 0.043-0.358) and TT (HR 8.988, CI 3.092-26.126) were significant factors (p<0.05). In the propensity-score matching cohort between TT and OST groups (**Table 6B**), the CCI group 1 (HR 1.736, CI 1.115-2.703) and CCI group 3 (HR 1.86, CI 1.23-2.812), regional SEER stage (HR 1.617, CI 1.178-2.22), and the time from nephrectomy to the secondary treatment since 2 years (24-35 months, HR 0.261 CI 0.148-0.459; 36-month< HR 0.114, CI 0.066-0.198) were significant factors (p<0.05).

**Table 6:** Multivariate analysis of cancer specific survival in 1:1 PSM matched RCC patients between (A) OP and TT groups and (B) TT and OST groups with postoperative disease progression after nephrectomy.

(A)								
	Univariate				Multivariate^1)^			
	HR	95% CI		p	HR	95% CI		p
Sex								
Male	1							
Female	1.533	0.754	3.12	0.2382				
Age at cancer diagnosis, year								
19–39	1							
40–49	0.692	0.286	1.674	0.4145				
50–59	0.439	0.174	1.106	0.0808				
60–69	0.319	0.086	1.179	0.0867				
70+	0.6	0.076	4.738	0.6277				
Residence								
Rural	1							
Urban	1.558	0.787	3.087	0.2034				
BMI								
Underweight	1							
Normal	1.604	0.208	12.348	0.6499				
Obese	0.304	0.028	3.351	0.3307				
Very obese	1.876	0.17	20.728	0.6077				
Comorbidities								
HTN	0.864	0.389	1.919	0.7193				
DM	0.586	0.293	1.173	0.1314				
Cardiovascular disease	0.499	0.232	1.075	0.076	0.474	0.179	1.255	0.1328
Cerebrovascular disease	0.194	0.027	1.424	0.1069				
CCI								
0	1							
1	0.754	0.287	1.983	0.5676				
2	0.553	0.189	1.619	0.2798				
3	1.357	0.575	3.199	0.4856				
Histology								
Non-clear cell	1				1			
Clear cell	0.196	0.047	0.823	0.026	0.265	0.049	1.429	0.1223
Insurance								
Occupational	1							
Regional	1.236	0.62	2.466	0.5475				
Medicare	NS							
Seer Summary stage								
Localized	1							
Regional	2.066	0.797	5.355	0.1355				
Year of cancer diagnosis								
2007	1							
2008	1.062	0.404	2.792	0.9026				
2009	0.542	0.158	1.866	0.3318				
2010	0.822	0.236	2.866	0.7581				
2011	1.228	0.337	4.477	0.7557				
2012	1.812	0.429	7.657	0.4186				
2013	0	0	.	0.9886				
Time from cancer diagnosis to first op								
0 days	1							
1–7 days	0.935	0.337	2.597	0.8978				
8–14 days	0.664	0.191	2.312	0.5199				
15–21 days	0.492	0.141	1.715	0.2655				
30+ days	1.613	0.672	3.871	0.2846				
Time from nephrectomy to the secondary treatment for disease recurrence								
~9 months	1				1			
10–11 months	1.569	0.341	7.229	0.5632	2.046	0.348	12.038	0.4285
12–23 months	0.751	0.318	1.773	0.5135	0.559	0.228	1.369	0.2034
24–35 months	0.315	0.099	1.006	0.0512	0.24	0.073	0.784	0.0182
36+ months	0.144	0.052	0.401	0.0002	0.124	0.043	0.358	0.0001
Second treatment type								
Surgery	1				1			
Targeted therapy	8.447	2.956	24.144	<.0001	8.988	3.092	26.126	<.0001
(B)								
	Univariate				Multivariate^1)^			
	HR	95% CI		p	HR	95% CI		p
Sex								
Male	1							
Female	1.101	0.786	1.542	0.5756				
Age at cancer diagnosis, y								
19–39	1							
40–49	1.201	0.44	3.28	0.7213				
50–59	0.883	0.341	2.288	0.7976				
60–69	1.189	0.476	2.969	0.711				
70+	1.115	0.446	2.786	0.8159				
Residence								
Rural	1							
Urban	0.976	0.718	1.328	0.8795				
BMI								
Underweight	1							
Normal	0.801	0.285	2.255	0.6747				
Obese	0.609	0.209	1.779	0.3648				
Very obese	0.258	0.029	2.316	0.2264				
Comorbidities								
HTN	1.124	0.748	1.69	0.5733				
DM	1.127	0.817	1.557	0.466				
Cardiovascular disease	0.727	0.52	1.018	0.0635				
Cerebrovascular disease	0.688	0.471	1.004	0.0525				
CCI								
0	1				1			
1	1.71	1.102	2.654	0.0166	1.736	1.115	2.703	0.0147
2	1.013	0.607	1.689	0.9617	0.924	0.553	1.543	0.762
3	1.541	1.025	2.316	0.0378	1.86	1.23	2.812	0.0033
Histology								
Non-clear cell	1							
Clear cell	0.919	0.377	2.238	0.8516				
Seer Summary stage								
Localized	1				1			
Regional	2.101	1.538	2.871	<.0001	1.617	1.178	2.22	0.003
Year of cancer diagnosis								
2007	1							
2008	0.759	0.463	1.245	0.2754				
2009	0.921	0.544	1.56	0.7605				
2010	0.989	0.582	1.679	0.9659				
2011	0.957	0.541	1.691	0.8789				
2012	1.074	0.58	1.99	0.8201				
2013	1.064	0.43	2.631	0.8931				
Time from cancer diagnosis to first op								
0 days	1							
1–7 days	0.814	0.486	1.362	0.4325				
8–14 days	1.287	0.796	2.081	0.3035				
15–21 days	0.761	0.475	1.22	0.2566				
30+ days	0.884	0.584	1.337	0.5582				
Time from nephrectomy to the secondary treatment for disease recurrence								
~9 months	1				1			
10–11 months	0.844	0.456	1.563	0.5905	0.8	0.429	1.492	0.483
12–23 months	0.645	0.449	0.927	0.0178	0.645	0.448	0.928	0.0181
24–35 months	0.25	0.142	0.437	<.0001	0.261	0.148	0.459	<.0001
36+ months	0.115	0.067	0.198	<.0001	0.114	0.066	0.198	<.0001
Second treatment type								
Other systemic agent therapy	1				1			
Targeted therapy	1.225	0.899	1.668	0.1983	1.24	0.909	1.693	0.1752

BMI was calculated as prediagnosis weight divided by adult height squared (kg/m^2^) and categorized according to the WHO criteria for Asian populations. A BMI between 18.6 and 22.9 kg/m^2^ was classified as normal, 23 to 24.9 kg/m^2^ as overweight, 25 to 29.9 kg/m^2^ as obese, and > 30 kg/m^2^ as severely obese.^22^

### Survival Comparisons Between Groups

The Kaplan-Meier OS/CSS curve showed a prominently better OS/CSS in the OP group than in other treatment groups (p<0.05, **Figure 2**). The median OS/CSS of the OP, TT, OST, and RT groups were 54.9/54.9, 41.7/41.9, 42.9/43.5, and 38.0/38.0 months, respectively (p<0.001, **Figure 2**). For radically nephrectomized RCC patients, the median OS of the OP, TT, OST, and RT groups were 55.3, 41.6, 40.9, and 27.7 months, respectively (p<0.001, **Supplementary Figure 1**).

## Discussion

This population-based retrospective cancer registry study is the first real-world descriptive and analytic study reporting the Asian therapeutic strategies and predictive risk factors for OS in recurrent loRCC following either radical or partial nephrectomy. Due to its low incidence rate (<5–7% for loRCC) and heterotrophic and heterogenic nature, it is not easy to design randomized clinical trials to predict disease recurrence of nephrectomized loRCC by comparing prognostic outcomes among therapeutic strategies and to generalize standardized therapeutic protocols for recurrent loRCC. Real clinical situations force many clinicians to depend upon their own experience and judgment for treatment decisions in recurrent loRCC, based on retrospective multicentric studies and small-sample case-cohort studies (1, 8, 11–13). This study provides clinical data for informing therapeutic decisions in recurrent loRCC after curative nephrectomy based on real-world outcomes from different therapeutic strategies. Moreover, this study identified that the time from nephrectomy to disease recurrence or secondary treatment for recurrent loRCC is an important prognostic factor for OS.

Various therapeutic treatments have been applied for recurrent loRCC after nephrectomy in combination with local or systemic therapies. Consistent with our findings, the best prognostic outcome was reported for surgical removal of the recurrent tumor to make the patient pathologically and radiologically tumor-free (1, 6, 7, 11, 13); the patients also had better outcomes with younger age, lower isolated tumor burden, and lesions that allowed for successful surgery under general anesthesia such as retroperitoneal lymph node at renal fossa, lung, or bone (11, 13, 14). Having a higher tumor burden with more-disseminated areas, a poorer general condition, and greater underlying disease that require non-surgical treatments for the disease recurrence each resulted in worse OS than that of the OP group.

Furthermore, minimally invasive surgical modalities, including laparoscopy and robot-assisted surgery (1, 6, 7, 15–17), have enabled successful surgery for low recurrent tumor burdens, with longer disease-free states to the next systemic therapy, fewer severe complications, earlier recovery, and allowing for interventive application of adjuvant systemic agent or focal therapy (2, 11, 12, 14, 17–19). Retroperitoneal lymph node dissection, venous thrombectomy, and cytoreductive metastasectomy of distant metastatic organs or perirenal fossa are the best surgical operations that allow better prognostic outcomes in recurrent loRCC (20, 21), in which patients recover earlier, with less-severe complications, and more-enhanced strategic management.

Unexpectedly, the RT group showed the worst OS (HR 7.47; **Table 3** and **Figure 2**). Since RCCs are radioresistant tumors, most recurrent cases received radiation therapy as adjuvant therapy combined with other surgical or systemic agent therapies. However, the RT group comprises cases that received only RT since they were unsuitable for either surgery or systemic therapies due to older age, poorer general condition, greater underlying disease, and higher tumor burdens (**Table 2**). The radiation therapy in the RT group was performed for palliative rather than the curative purpose and specific recurrent lesions related to high morbidities, such as central nervous system and/or bone lesions. Systemic or external beam radiation therapy targeted at the localized site is reported to have a 5-year OS of 40–73% for recurrent RCC after nephrectomy (18, 19, 21). This might explain why the RT group had the worst OS and a higher rate of non-RCC deaths.

The systemic-agent groups in this study, OST (HR 7.05 for OS) and TT (HR 6.27), were composed of inoperable patients, with insignificant differences between them, and a worse OS is expected compared to the OP group. Since the introduction of TT agents in 2005, the survival of RCC has improved markedly by a median 6–18 months, as compared to cytokine usage for advanced or metastatic RCC in the pre-TT era. Treatment with only OST agents indicated only a small portion of specific histological RCC in the TT era (25.79% for OST *vs.* 59.46% for TT); therefore, it is not easy to compare the OS between the TT and OST groups. Diverse clinical trials using TT agents have been testified and are ongoing as neoadjuvant and adjuvant settings; they failed to provide survival benefits, and adverse events increased in loRCC cohorts after nephrectomy, except for some highly selected high-risk patients (1, 6, 15). However, some positive effects of systemic TT and OST were identified in trials, showing significant downsizing of the tumor burden, decreasing local recurrence or metastasis, and allowing other subsequent treatments (1, 6, 15, 16). Although this study did not include recently introduced immune-checkpoint inhibitors, a combined TT and OST agent might further improve OS without severe adverse events in recurrent loRCC, or in the prevention of disease recurrence, in future neoadjuvant and adjuvant trials (9, 15, 22, 23).

Four significantly favorable predictive factors of OS have been identified: the female sex (HR: 0.72) (24), a regional SEER stage (HR: 1.74), a longer period between nephrectomy and post-nephrectomized secondary treatments after one postoperative year (HR: <1.0 y) (25–27), and the secondary treatment types (HR: >6.0 for TT, OST, and RT) (p<0.05; **Table 3** and **Supplementary Table 2**). Significantly, an interval between nephrectomy and disease recurrence of less than one year is already a proven factor of unfavorable survival prognosis in metastatic and advanced RCC (25–27). The disease recurrence within one year after nephrectomy was affected most by poorer baseline general conditions and poorer pathologic characteristics of the primary loRCC, suggesting that invisible circulating tumors or an inherent dormant metastatic niche might have already existed at the time of nephrectomy despite a free resection margin. Therefore, further studies would be needed to expand the indications for active adjuvant therapies and identify a patient’s baseline general conditions, including immune status and pathologic information on the primary tumor.

Since about half of the disease recurrences occur within 2 years after nephrectomy, and this study showed that one-year postoperative treatment interval was a significant factor for favorable OS (p<0.05, HR: 0.61 for 13–24 months, HR: 0.33 for 25–36 months and HR: 0.12 for > 36 months, **Table 3**), it is important to discuss the benefit of early secondary treatment for recurrent loRCC between 1–2 years after nephrectomy. Furthermore, since no postoperative follow-up biomarkers, no adequate indicators for surgical timing of metastasectomy or local surgery, and no effective adjuvant therapies have been established in recurrent loRCC (1, 4–7), an intensive conditional postoperative plan and detection protocol should be developed using imaging and blood biomarkers within 1–2 years and afterward (9, 11, 28). For example, in male patients with regional SEER staging, it is important to consider a postoperative follow-up plan within two years using more systemic imaging modalities and more additional prediction biomarkers. The PET-CT and personalized liquid biopsies for blood PD-1 and genetic biomarkers have not been approved as earlier detecting modalities of disease recurrence with a lower tumor burden or isolated tumor recurrence (29, 30). However, such additional modalities within postoperative two years might allow for surgical removal of the targeted recurrent lesion as early as possible, since many clinicians wait for the appearance of recurrent tumor lesion greater than 1 cm confirmed on currently recommended CT imaging modalities (1, 2, 5, 7).

This study had inherent limitations, including its retrospective design and the absence of data for radiological information to estimate the extent of involved organ and site-related tumor burdens, neoadjuvant and adjuvant purposes, the sequential combination of major secondary therapies with the lead-time bias effect, and organ and sites of disease recurrence. However, this is the first study in Korea describing the therapeutic trends of recurrent loRCC after nephrectomy, their survival outcomes, and significant predictive factors. Further prospective, large-scale, longitudinal studies are needed to evaluate disease recurrence for different multi-treatment therapies, including the survival outcomes, intervention measures, and different drug mechanisms.

## Conclusion

This population-based retrospective study showed several significant risk factors affecting OS in recurrent loRCC following nephrectomy. Surgery had the highest OS as a secondary therapeutic option for disease recurrence, whereas TT and OST showed insignificant OS differences. The importance of the disease-free period following nephrectomy suggests that an intensive postoperative follow-up protocol should be evaluated for improving the prognostic outcomes of disease recurrence in loRCC.

## Data Availability Statement

The original contributions presented in the study are included in the article/**Supplementary Material**. Further inquiries can be directed to the corresponding authors.

## Ethics Statement

The studies involving human participants were reviewed and approved by National Cancer Center. Written informed consent for participation was not required for this study in accordance with the national legislation and the institutional requirements.

## Author Contributions

SK: Conceptualization, Data Curation, Investigation, Methodology, Project Administration, Supervision and Writing – Original Draft Preparation. YK, MC, and JS: Conceptualization, Data Curation, Formal Analysis, Investigation, Methodology, Project Administration, Supervision and Writing – Original Draft Preparation. JC: Conceptualization, Data Curation, Investigation, Methodology, Project Administration, Supervision, Funding Acquisition and Writing – Original Draft Preparation. All authors contributed to the article and approved the submitted version.

## Funding

This work was supported by a grant from the National Cancer Center (No. 1610310, 1810021, and 1910171), Republic of Korea. This work was supported by a grant from the Korean Urological Oncology Association in 2019.

## Conflict of Interest

The authors declare that the research was conducted in the absence of any commercial or financial relationships that could be construed as a potential conflict of interest.

## Publisher’s Note

All claims expressed in this article are solely those of the authors and do not necessarily represent those of their affiliated organizations, or those of the publisher, the editors and the reviewers. Any product that may be evaluated in this article, or claim that may be made by its manufacturer, is not guaranteed or endorsed by the publisher.
