# Supplementary material for: A Real-World, Population-Based Retrospective Analysis of Therapeutic Survival for Recurrent Localized Renal Cell Carcinoma After Nephrectomy
Source: Front Oncol. 2021 Sep 8;11:693831. doi: 10.3389/fonc.2021.693831 (PMC8456083; doi:10.3389/fonc.2021.693831)
Supplement: Supplementary file 1 [file DataSheet_1.docx]

Supplementary Table 1. Baseline characteristics of radically or partially nephrectomized recurrent localized RCC patients

|  | 1st TX | | | | |
| --- | --- | --- | --- | --- | --- |
|  | partial nephrectomy (n=171) | | radical nephrectomy (n=1,124) | | *p-value* |
|  | n | % | n | % |  |
| Sex |  |  |  |  |  |
| Male | 139 | 81.3 | 856 | 76.2 | 0.139 |
| Female | 32 | 18.7 | 268 | 23.8 |  |
| Age at cancer diagnosis, y |  |  |  |  |  |
| 19–39 | 12 | 7 | 61 | 5.4 | 0.588 |
| 40–49 | 17 | 9.9 | 163 | 14.5 |  |
| 50–59 | 47 | 27.5 | 305 | 27.1 |  |
| 60–69 | 57 | 33.3 | 334 | 29.7 |  |
| 70–79 | 34 | 19.9 | 228 | 20.3 |  |
| 80+ | 4 | 2.3 | 33 | 2.9 |  |
| Residence |  |  |  |  |  |
| Rural | 93 | 54.4 | 577 | 51.3 | 0.457 |
| Urban | 78 | 45.6 | 547 | 48.7 |  |
| Insurance |  |  |  |  |  |
| Occupational | 112 | 65.5 | 684 | 60.9 | 0.120 |
| Regional | 55 | 32.2 | 372 | 33.1 |  |
| Medicare | 4 | 2.3 | 68 | 6.1 |  |
| Seer Summary stage |  |  |  |  |  |
| Localized | 161 | 94.2 | 698 | 62.1 | <0.001 |
| Regional | 10 | 5.9 | 426 | 37.9 |  |
| Year of cancer diagnosis |  |  |  |  |  |
| 2007 | 21 | 12.3 | 174 | 15.5 | 0.620 |
| 2008 | 22 | 12.9 | 174 | 15.5 |  |
| 2009 | 24 | 14 | 162 | 14.4 |  |
| 2010 | 31 | 18.1 | 189 | 16.8 |  |
| 2011 | 24 | 14 | 159 | 14.2 |  |
| 2012 | 24 | 14 | 150 | 13.4 |  |
| 2013 | 25 | 14.6 | 116 | 10.3 |  |
| Time from cancer diagnosis to first op | |  |  |  |  |
| 0 days | 54 | 31.6 | 379 | 33.7 | 0.944 |
| 1–7 days | 20 | 11.7 | 141 | 12.5 |  |
| 8–14 days | 25 | 14.6 | 156 | 13.9 |  |
| 15–21 days | 35 | 20.5 | 231 | 20.6 |  |
| 30+ days | 37 | 21.6 | 217 | 19.3 |  |
| time from nephrectomy to the secondary treatment for disease recurrence | | | | | |
| ~9 months | 45 | 26.3 | 302 | 26.9 | 0.312 |
| 10–12 months | 4 | 2.3 | 60 | 5.3 |  |
| 13–24 months | 37 | 21.6 | 277 | 24.6 |  |
| 25–36 months | 32 | 18.7 | 177 | 15.8 |  |
| 37+ months | 53 | 31 | 308 | 27.4 |  |
| Second treatment type | | | | | |
| Surgery | 58 | 33.9 | 97 | 8.6 | <0.001 |
| Targeted therapy | 40 | 23.4 | 730 | 65 |  |
| Other systemic therapy | 66 | 38.6 | 268 | 23.8 |  |
| Radiotherapy | 7 | 4.1 | 29 | 2.6 |  |
| Mortality |  |  |  |  |  |
| Alive | 140 | 81.9 | 734 | 65.3 | <0.001 |
| Death | 31 | 18.1 | 390 | 34.7 |  |
| *Cancer cause death* | *11* | *35.5* | *36* | *9.2* | *<0.001* |
| *Other cancer cause death* | *18* | *58.1* | *329* | *84.4* |  |
| *Other cause death* | *2* | *6.5* | *25* | *6.4* |  |

Supplementary table 2. Multivariate analysis of overall survival among 1124 radical nephrectomized RCC patients with recurrence

|  | Crude | | | |  | Adjusted | | | |
| --- | --- | --- | --- | --- | --- | --- | --- | --- | --- |
|  | HR | 95% CI | | p |  | HR | 95% CI | | p |
| Sex |  |  |  |  |  |  |  |  |  |
| Male | 1 |  |  |  |  | 1 |  |  |  |
| Female | 0.95 | 0.75 | 1.20 | 0.635 |  | 0.74 | 0.58 | 0.95 | 018 |
| Age at cancer diagnosis, y |  |  |  |  |  |  |  |  |  |
| 19–39 | 1 |  |  |  |  | 1 |  |  |  |
| 40–49 | 0.93 | 0.56 | 1.55 | 0.777 |  | 0.93 | 0.55 | 1.56 | 0.773 |
| 50–59 | 0.75 | 0.46 | 1.21 | 0.236 |  | 0.62 | 0.38 | 11 | 0.054 |
| 60–69 | 1.18 | 0.74 | 1.87 | 0.491 |  | 0.97 | 0.60 | 1.57 | 0.905 |
| 70–79 | 1.43 | 0.89 | 2.30 | 0.140 |  | 1.21 | 0.74 | 1.97 | 0.442 |
| 80+ | 1.84 | 0.97 | 3.49 | 062 |  | 1.49 | 0.77 | 2.88 | 0.241 |
| Residence |  |  |  |  |  |  |  |  |  |
| Rural | 1 |  |  |  |  | 1 |  |  |  |
| Urban | 16 | 0.87 | 1.29 | 0.595 |  | 0.91 | 0.75 | 1.12 | 0.385 |
| Insurance |  |  |  |  |  |  |  |  |  |
| Occupational | 1 |  |  |  |  | 1 |  |  |  |
| Regional | 16 | 0.86 | 1.31 | 0.604 |  | 0.92 | 0.74 | 1.15 | 0.456 |
| Medicare | 0.97 | 0.62 | 1.52 | 0.902 |  | 0.99 | 0.63 | 1.57 | 0.977 |
| Seer Summary stage |  |  |  |  |  |  |  |  |  |
| Localized | 1 |  |  |  |  | 1 |  |  |  |
| Regional | 21 | 1.64 | 2.45 | <001 |  | 1.48 | 1.19 | 1.83 | <0.001 |
| Year of cancer diagnosis |  |  |  |  |  |  |  |  |  |
| 2007 | 1 |  |  |  |  | 1 |  |  |  |
| 2008 | 1.11 | 0.81 | 1.51 | 0.527 |  | 1.211 | 0.88 | 1.67 | 0.244 |
| 2009 | 1.12 | 0.81 | 1.56 | 0.500 |  | 1.13 | 0.80 | 1.59 | 0.485 |
| 2010 | 1.11 | 0.79 | 1.55 | 0.562 |  | 0.886 | 0.62 | 1.26 | 0.502 |
| 2011 | 1.30 | 0.89 | 1.89 | 0.170 |  | 0.95 | 0.65 | 1.40 | 0.795 |
| 2012 | 1.39 | 0.90 | 2.13 | 0.135 |  | 0.90 | 0.58 | 1.40 | 0.633 |
| 2013 | 1.19 | 0.62 | 2.29 | 0.607 |  | 0.64 | 0.33 | 1.24 | 0.185 |
| Time from cancer diagnosis to nephrectomy | | |  |  |  |  |  |  |  |
| 0 days | 1 |  |  |  |  | 1 |  |  |  |
| 1–7 days | 0.89 | 0.64 | 1.24 | 0.501 |  | 0.81 | 0.57 | 1.14 | 0.216 |
| 8–14 days | 0.84 | 0.60 | 1.17 | 0.304 |  | 0.85 | 0.60 | 1.20 | 0.354 |
| 15–21 days | 0.88 | 0.66 | 1.16 | 0.359 |  | 0.87 | 0.65 | 1.16 | 0.352 |
| 30+ days | 14 | 0.80 | 1.37 | 0.758 |  | 0.82 | 0.62 | 18 | 0.165 |
| Time from nephrectomy to secondary treatment for disease recurrence | | |  |  |  |  |  |  |  |
| ~9 months | 1 |  |  |  |  | 1 |  |  |  |
| 10–12 months | 11 | 0.69 | 1.47 | 0.975 |  | 0.98 | 0.66 | 1.45 | 0.913 |
| 13–24 months | 0.60 | 0.47 | 0.77 | <001 |  | 0.56 | 0.43 | 0.72 | <0.001 |
| 25–36 months | 0.31 | 0.22 | 0.43 | <001 |  | 0.28 | 0.20 | 0.39 | <0.001 |
| 37+ months | 0.11 | 08 | 0.15 | <001 |  | 0.11 | 08 | 0.15 | <0.001 |
| Second treatment type |  |  |  |  |  |  |  |  |  |
| Surgery | 1 |  |  |  |  | 1 |  |  |  |
| Targeted therapy | 7.40 | 3.29 | 16.63 | <001 |  | 5.60 | 2.46 | 12.74 | <0.001 |
| Other systemic therapy | 9.18 | 44 | 20.87 | <001 |  | 6.25 | 2.71 | 14.40 | <0.001 |
| Radiotherapy | 9.83 | 3.63 | 26.58 | <001 |  | 6.78 | 2.46 | 18.72 | 0.001 |

Supplementary Figure 1. Kaplan-Meier plot of overall survival according to the therapeutic groups among radical nephrectomized patients with recurrence


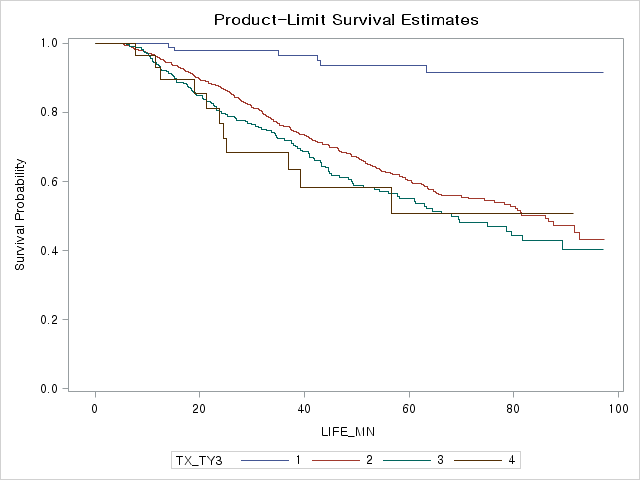


|  | OS time (months) | | | | |
| --- | --- | --- | --- | --- | --- |
|  | **mean** | **SD** | **min** | **max** | **median** |
| 1. OP | 56.9 | 24.4 | 12.4 | 97.2 | 55.3 |
| 2. TT | 45.3 | 23.9 | 5.3 | 97.2 | 41.6 |
| 3. OST | 45 | 24.8 | 6 | 97.1 | 40.9 |
| 4. RT | 38.4 | 25.2 | 7.6 | 91.4 | 27.7 |

OP=surgery, TT=targeted therapy, OST=other systemic agent therapy, RT=radiotherapy
